# Design, Synthesis and Evaluation of Antitubercular Activity of Novel Dihydropyridine Containing Imidazolyl Substituent 

**Published:** 2015

**Authors:** Maryam Iman, Asghar Davood, Golnoush Dehqani, Mahboubeh Lotfinia, Soroush Sardari, Parisa Azerang, Mohsen Amini

**Affiliations:** a *Chemical Injuries Research Center, Baqiyatallah University of Medical Sciences, Tehran, Iran.*; b *Department of Medicinal Chemistry, Faculty of Pharmacy, Pharmaceutical Sciences Branch, Islamic Azad University, Tehran, Iran (IAUPS). *; c *Department of Bioinformatics and Drug design, Institute Pasteur, Tehran, Iran. *; d *Department of Medicinal Chemistry, Faculty of Pharmacy, Tehran University of Medical Sciences, Tehran, Iran.*

**Keywords:** Dihydropyridine, Imidazole, Mycobacterium, Synthesis, Tuberculosis

## Abstract

Recent studies have indicated that 1, 4-dihydropyridine-3, 5-dicarboxamide derivatives show significant anti-tubercular activity. In this research, new derivatives of 1, 4-dihydropyridine were designed and synthesized using Hantzsch condensation in which dicyclohexyl and different dicyclohexylcarbamoyl were substituted at C-3 and C-5 positions of the DHP ring. In addition, 4 (5)-chloro-2-methyl-5 (4)-imidazolyl moiety was substituted at C-4 position of DHP. The structure of synthetized compounds were characterized by TLC, IR, elemental analysis and proton NMR. Based on the in vitro screening data, all of the designed and synthetized compounds (3a-3g) showed a good ability to inhibit the mycobacterium tuberculosis growth in terms of MIC. Aromatic carboxamide containing compounds were more potent than cyclohexyl derivative and the most potent compound was 3a (4-nitrophenyl derivative). The experimental data are in agreement with our computational predictions in terms of partial atomic charge of carbonyl moieties at the C-3 and C-5 positions of DHP ring and partition coefficient of the molecules.

## Introduction

1, 4-Dihydropyridine (DHP) is a multifunctional lead molecule and acts as a calcium channel modulator (1-6). The feasible positions for substitution are 3, 4 and 5 which exhibit various pharmacological activities such as antihypertensive (7-8), anticancer (9), MDRr (10-12), antianginal (7-8), antitubercular (9, 13-16), antioxidant (17-18), analgesic and antiinflammatory (19), antithrombotic (20-21), anticonvulsant (22-23), stress protective (24), antimicrobial (9), antidyslipidemic (18) and antiulcer (25).

**Figure 1:**
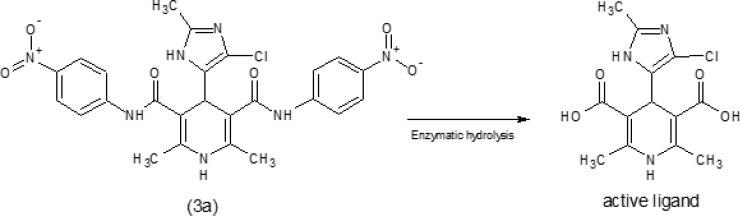
Enzymatic bio-activation of dihydropyridines

Based on the MDRr (10-12) and anti-tubercular (9, 13-16) activities of DHPs, it seems DHPs is excellent lead compound to find and develop of novel anti-tubercular agents. It was confirmed that the replacement of the dicarboxylic esters group of DHPs with the aryl amide (carboxamide) moiety, reduces the calcium channel antagonist activity and increases the anti-tubercular potency (26). It was suggested that DHPs with dicarboximide moieties may act as precursors and after penetration into the mycobacterium cell wall; carboximide groups may undergo enzymatic hydrolysis (27) and convert to carboxylic acid moiety (Figure 1). So it seems two factors; lipophilicity (log p) of molecule and partial atomic charge (PAC) of carbon atom of carbonyl at C-3 and C-5 positions of DHPs are important in antitubercular activity because of their effect on the penetration and enzymatic bio-activation of DHPs respectively.

**Figure 2:**
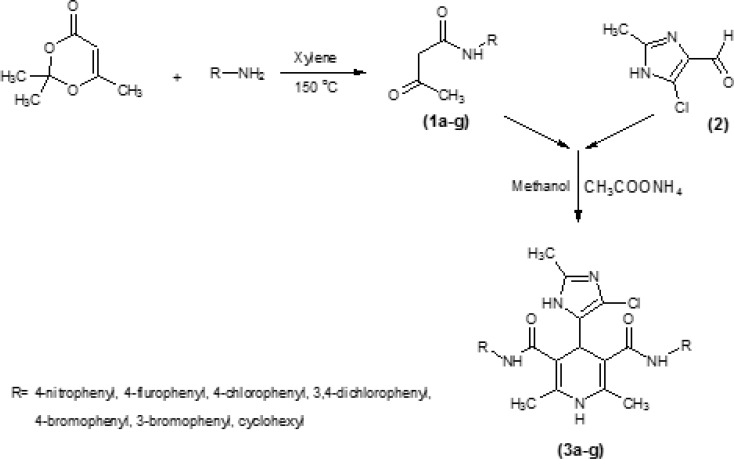
Synthesis of symmetrical dicarboxamide 3a-g, by using classical Hantzsch condensation.

In our previous studies we confirmed that in DHPs, 4 (5)-chloro-2-methyl-5 (4)-imidazolyl substituent is bioisoster of nitrophenyl in nifedipine (3-6). Here in as a part of our ongoing research to design and synthesis of new DHPs and based on the above mentioned subject, using 4 (5)-chloro-2-methyl-5 (4)-imidazolyl and aryl amide moieties in the C-4, C-3 and C-5 of DHP ring, some novel DHPs were designed, synthetized and evaluated as antitubercular agents. 

## Experimental

*Chemistry*

Reagents and solvents were obtained from MERCK (Darmstadt, Germany). All of the compounds were characterized by TLC, IR, elemental analysis and proton NMR. Melting points were determined using a Thomas- Hoover capillary apparatus and were uncorrected. ^1^HNMR spectra were recorded on a Bruker FT-500 spectrometer and TMS was used as an internal standard. Infrared spectra were acquired on a Nicolet 550-FT spectrometer. Elemental analysis was carried out with a Perkin-Elmer model 240-C apparatus. The results of elemental analysis (C, H, and N) were within ± 0.4% of the calculated amounts.

Symmetrical dicarboxamides 3a-g (Table 1) were synthesized according to Figure 2, by using classical Hantzsch condensation (28-29) in which 4 (5)-chloro-2-methylimidazole-5 (4)-carboxaldehyde 2 was reacted with N-arylacetoacetamides 1a-g and ammonium acetate in methanol. The compound 2 could be prepared in three-step from acetaldehyde, dihydroxy acetone and ammonia (6). N-arylacetoacetamides 1a-g was synthesized according to modified Clemens method (30) by condensation of 2, 2, 6-trimethyl-1, 3-dioxin-4-one with the appropriate arylamines.

**Table 1:** Structure and *in-vitro* anti-tubercular activity of compounds 3a-g.

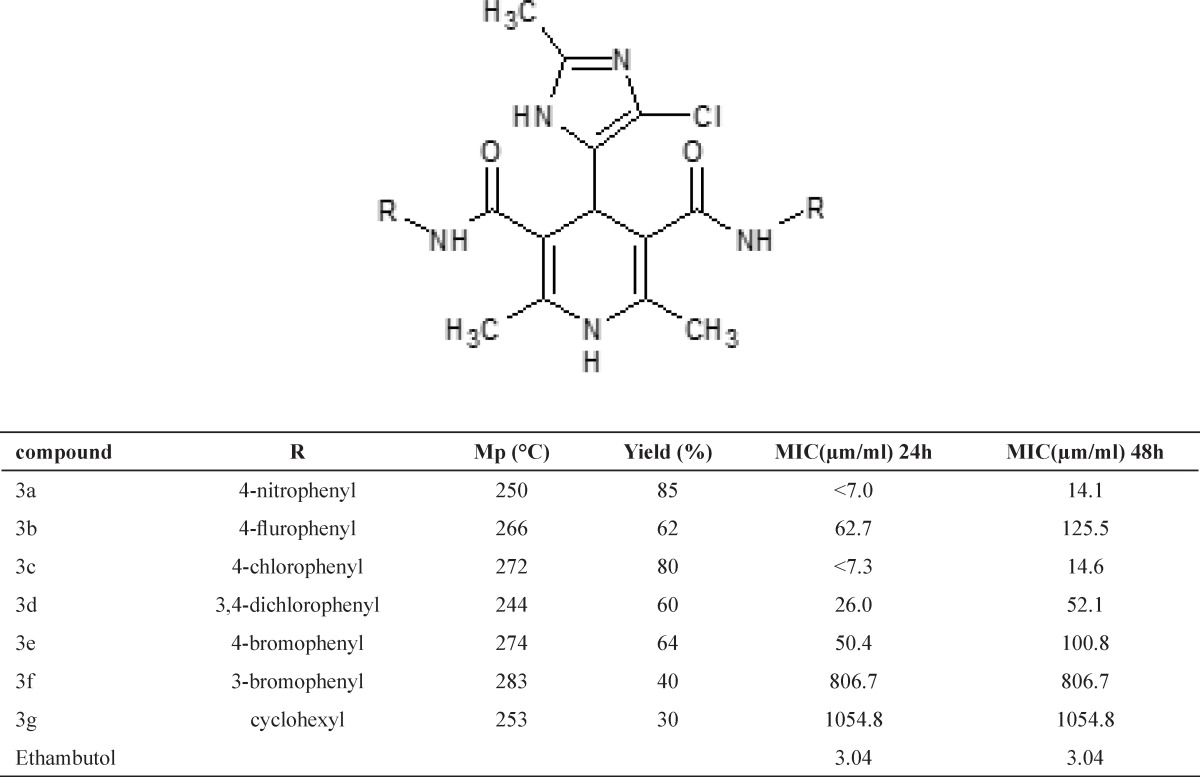

*General procedure for the prepartation of 3-oxo-N-aryl (alkyl) butanamide (1a-g)*

A solution of an amine and dioxin in xylene was placed in an Erlenmeyer flask. The flask was immersed into an oil bath that had been preheated to 150°C, and the solution was vigorously stirred. The evolution of acetone became apparent within several minutes and heating was continued for around 4 h. The xylene was then removed, and the product was filtered and recrystallized from appropriate organic solvents.

*N-(4-nitrophenyl)-3-oxobutanamide (1a)*

Using the general procedure and 4-nitroaniline provided the title compound after 4 h of reflux: Yellow crystals, Yield 70%, mp 142-143°C. IR (KBr): ν 3325(NH), 1719, 1685 cm^−1^ (CO). ^1^H-NMR (CDCl_3_): δ 9.70 (brs, 1H, NH), 8.20 (d, J = 8.8 Hz, 2H, H-3, 5-phenyl), 7.80 (d, J = 8.8 Hz, 2H, H-2, 6-phenyl), 3.65 (s, 2H, CH_2_), 2.30 ppm (s, 3H, CH_3_).

*N-(4-fluorophenyl)-3-oxobutanamide (1b)*

Using the general procedure and 4-fluoroaniline provided the title compound after 3 h of reflux: White crystals, Yield 88%, mp 98-101°C. IR (KBr): ν 3315(NH), 1716, 1671 cm^−1^ (CO). ^1^H-NMR (CDCl_3_): δ 9.40 (brs, 1H, NH), 7.56-7.78 (m, 2H, H-2, 6-phenyl), 7.15-7.25 (m, 2H, H-3, 5-phenyl), 3.70 (s, 2H, CH_2_), 2.29 ppm (s, 3H, CH_3_).

*N-(4-chlorophenyl)-3-oxobutanamide (1c)*

Using the general procedure and 4-chloroaniline provided the title compound after 3 h of reflux: White crystals, Yield 79%, mp 127-129°C. IR (KBr): ν 3300 (NH), 1720, 1670 cm^−1^ (CO). ^1^H-NMR (CDCl_3_): δ 9.37 (s, 1H, NH), 7.50 (d, J = 8 Hz, 2H, aromatic), 7.23 (d, J = 8 Hz, 2H, aromatic), 3.58 (s, 2H, CH_2_), 2.30 ppm (s, 3H, CH_3_).

*N-(3, 4-dichlorophenyl)-3-oxobutanamide (1d)*

Using the general procedure and 3, 4-dichloroaniline provided the title compound after 4 h of reflux: White crystals, Yield 65%, mp 79-81°C. IR (KBr): ν 3378 (NH), 1710, 1665 cm^−1^ (CO). ^1^H-NMR (CDCl_3_): δ 9.45 (brs, 1H, NH), 8.05 (s, 1H, H-2-phenyl), 7.30-7.47 (m, 2H, H-5, 6-phenyl), 3.65 (s, 2H, CH_2_), 2.39 ppm (s, 3H, CH_3_).

*N-(4-bromophenyl)-3-oxobutanamide (1e)*

Using the general procedure and 4-bromoaniline provided the title compound after 3 h of reflux: Light yellow crystals, Yield 75%, mp 135-136°C. IR (KBr): ν 3260 (NH), 1716, 1680 cm^−1^ (CO). ^1^H-NMR (CDCl_3_): δ 9.65 (brs, 1H, NH), 7.62 (d, J = 7.8 Hz, 2H, aromatic), 7.40 (d, J = 7.8 Hz, 2H, aromatic), 3.65 (s, 2H, CH_2_), 2.28 ppm (s, 3H, CH_3_).

*N-(3-bromophenyl)-3-oxobutanamide (1f)*

Using the general procedure and 3-bromoaniline provided the title compound after 3 h of reflux: Light yellow crystals, Yield 80%, mp 98-101°C. IR (KBr): ν 3300 (NH), 1712, 1673 cm^−1^ (CO). ^1^H-NMR (CDCl_3_): δ 9.25 (brs, 1H, NH), 7.78 (s, 1H, H-2-phenyl), 7.18-7.45 (m, 3H, H-4, 5, 6-phenyl), 3.54 (s, 2H, CH_2_), 2.29 ppm (s, 3H, CH_3_).

*N-(cyclohexyl)-3-oxobutanamide (1g)*

Using the general procedure and cyclohexylamine provided the title compound after 3 h of reflux: Light yellow crystals, Yield 69%, mp 98-100°C. IR (KBr): ν 3310(NH), 1710, 1665 cm^−1^ (CO). ^1^H-NMR (CDCl_3_): δ 9.40 (brs, 1H, NH), 3.65 (s, 2H, CH_2_), 2.15-2.40 (m, 4H, CH_3_, and H-1-cyclohexyl), 1.30-1.75 ppm (m, 10H, cyclohexyl).

*General procedure for preparation of diaryl (cycloalkyl) 4-(4 (5)-chloro-1H-imidazol-5 (4)-yl)-2, 6-dimethyl-1, 4-dihydropyridine-3, 5-dicarboxamide (3a-g)*

A solution of compound 2 (150 mg, 1.038 mmol), ammonium acetate (80 mg, 1.038 mmol), and compounds 1a-g (2.076 mmol) in methanol (3 mL) was refluxed. The solvent was removed under reduced pressure and the residue was crystallized from appropriate solvent to give the title compounds.

*4-(4 (5)-chloro-2-methyl-1H-imidazol-5 (4)-yl)-2, 6-dimethyl-N* ^3^ *, N* ^5^ *-bis (4-nitrophenyl)-1, 4-dihydropyridine-3, 5-dicarboxamide (3a)*

Compound 3a was provided from compound 1a using general procedure after 35 h reflux: Yellow crystals, yield 85%; mp 250^o^C (ethyl acetate-methanol). IR (KBr): ν 3257 (NH), 1680, 1645 (CO), 1501, 1321 cm^−1^ (NO_2_). ^1^H-NMR (CDCl_3 _+ DMSO- *d*6): δ 2.27 (s, 9H, C-2,6-CH_3_ and CH_3_-imidazole), 5.25 (brs, 1H, H-4-DHP), 7.61 (s, 1H, NH-imidazole), 7.82 (d, J = 9.6 Hz, 4H, H-3ʹ,5ʹ-phenyl), 8.13 (d, J = 9.6 Hz, 4H, H-2ʹ,6ʹ-phenyl). 8.6 (s, 1H, NH-DHP), 9.4 ppm (br, 2H, NH-amide). Molecular formula: C_25_H_22_ClN_7_O_6_; Calculated = C (54.40%) H (4.02%) N (17.76%); Found = C (54.49%) H (4.03%) N (17.79%).

*4-(4 (5)-chloro-2-methyl-1H-imidazol-5 (4)-yl)-2, 6-dimethyl-N* ^3^ *, N* ^5^ *-bis (4-fluorophenyl)-1, 4-dihydropyridine-3, 5-dicarboxamide (3b)*

Compound 3b was provided from compound 1b using general procedure after 38 h reflux: White crystals, yield 62%; mp 266^o^C (methanol). IR (KBr): ν 3436, 3293, 3216 (NH), 1685, 1634 cm^−1^ (CO). ^1^H-NMR (CDCl_3 _+ DMSO- *d*6): δ 2.21 (s, 6H, C-2,6-CH_3_), 2.30 (s, 3H, CH_3_-imidazole), 5.19 (s, 1H, H-4-DHP), 6.94 (t, 5.4 Hz, 8.7 Hz, 4H, H-3ʹ,5ʹ-phenyl), 7.49-7.7 (m, 4H, H-2ʹ,6ʹ-phenyl), 8.10 (s, 1H, NH-DHP), 9.4 ppm (s, 2H, NH-amide). Molecular formula: C_25_H_22_ClF_2_N_5_O_2_; Calculated = C (60.30%) H (4.45%) N (14.07%); Found = C (60.37%) H (4.46%) N (14.05%).

*4-(4(5)-chloro-2-methyl-1H-imidazol-5(4)-yl)-2, 6-dimethyl-N* ^3^ *, N* ^5^ *-bis (4-chlorophenyl)-1, 4-dihydropyridine-3, 5-dicarboxamide (3c)*

Compound 3c was provided from compound 1c using general procedure after 30 h reflux: White crystals, yield 80%; mp 272^o^C (methanol). IR (KBr): ν 3445, 3398 (NH), 1676, 1661 cm^−1^ (CO). ^1^H-NMR (DMSO- *d*6): δ 2.01 (s, 6H, C-2,6-CH_3_), 2.11 (s, 3H, CH_3_-imidazole), 5.15 (s, 1H, H-4-DHP), 7.28 (d, 6.29 Hz, 4H, aromatic), 7.6 (d, J = 6.40 Hz, 4H, aromatic), 8.38 (s, 1H, NH-DHP), 9.3 ppm (s, 2H, NH-amide). Molecular formula: C_25_H_22_Cl_3_N_5_O_2_; Calculated = C (56.57%) H (4.18%) N (13.19%); Found = C (56.62%) H (4.17%) N (13.22%).

*4-(4 (5)-chloro-2-methyl-1H-imidazol-5 (4)-yl)-2, 6-dimethyl-N* ^3^ *, N* ^5^ *-bis (3, 4- dichlorophenyl)-1, 4-dihydropyridine-3, 5-dicarboxamide (3d)*

Compound 3d was provided from compound 1d using general procedure after 40 h reflux: White crystals, yield 60%; mp 244^o^C (methanol-ethyl acetate). IR (KBr): ν 3431, 3365 (NH), 1664 cm^−1^ (CO). ^1^H-NMR (CDCl_3 _+ DMSO- *d*6): δ 2.13 (s, 9H, C-2,6-CH_3 _and CH_3_-imidazole), 5.19 (s, 1H, H-4-DHP), 7.32 (m, 4H, H-5ʹ,6ʹ-phenyl), 7.68 (m, 2H, H-2ʹ-phenyl), 8.42 (s, 1H, NH-DHP), 9.44 ppm (brs, 2H, NH-amide). Molecular formula: C_25_H_20_Cl_5_N_5_O_2_; Calculated = C (50.07%) H (3.36%) N (11.68%); Found = C (50.04%) H (3.37%) N (11.71%).

*4-(4 (5)-chloro-2-methyl-1H-imidazol-5 (4)-yl)-2, 6-dimethyl-N* ^3^ *, N* ^5^ *-bis (4-bromophenyl)-1, 4-dihydropyridine-3, 5-dicarboxamide (3e)*

Compound 3e was provided from compound 1e using general procedure after 37 h reflux: White crystals, yield 64%; mp 274^o^C (methanol-ethyl acetate). IR (KBr): ν 3452, 3367 (NH), 1655 cm^−1^ (CO). ^1^H-NMR (CDCl_3 _+ DMSO- *d*6): δ 2.10 (s, 6H, C-2,6-CH_3_), 2.8 (s, 3H, CH_3_-imidazole), 5.11 (s, 1H, H-4-DHP), 7.08-7.28 (m, 4H, aromatic), 7.3-7.6 (m, 4H, aromatic), 8.33 (brs, 1H, NH-DHP), 9.31 ppm (s, 2H, NH-amide). Molecular formula: C_25_H_22_ Br_2_ClN_5_O_2_; Calculated = C (48.45%) H (3.58%) N (11.30%); Found = C (48.51%) H (3.59%) N (11.27%).

*4-(4(5)-chloro-2-methyl-1H-imidazol-5(4)-yl)-2, 6-dimethyl-N*^3^*, N*^5^*-bis (3-bromophenyl)-1, 4-dihydropyridine-3, 5-dicarboxamide (3f)*

Compound 3f was provided from compound 1f using general procedure after 38 h reflux: Green crystals, yield 40%; mp 283^o^C (methanol-ethyl acetate). IR (KBr): ν 3146(NH), 1649 cm^−1^ (CO). ^1^H-NMR (CDCl_3 _+ DMSO- *d*6): δ 2.10 (s, 6H, C-2, 6-CH_3_), 2.12 (s, 3H, CH_3_-imidazole), 5.11 (brs, 1H, H-4-DHP), 7.00-7.63 (m, 8H, aromatic), 8.35 (brs, 1H, NH-DHP), 9.31 ppm (s, 2H, NH-amide). Molecular formula: C_25_H_22_ Br_2_ClN_5_O_2_; Calculated = C (48.45%) H (3.58%) N (11.30%); Found = C (48.41%) H (3.59%) N (11.33%).

*4-(4(5)-chloro-2-methyl-1H-imidazol-5(4)-yl)-2, 6-dimethyl-N* ^3^ *, N* ^5^ *-bis (4-cyclohexyl)-1, 4-dihydropyridine-3, 5-dicarboxamide (3g)*

Compound 3g was provided from compound 1g using general procedure after 39 h reflux: Light yellow crystals, yield 30%; mp 253^o^C (ethyl acetate-methanol). IR (KBr): ν 3267 (NH), 2924, 2852 (CH-aliphatic), 1664 cm^−1^ (CO). ^1^H-NMR (CDCl_3_+DMSO- *d*6): δ 0.62-1.93 (m, 20H, Cyclohexyl), 1.97 (s, 6H, C-2, 6-CH_3_), 2.24 (s, 5H, CH_3_-imidazole and H-1-cyclohexyl), 5.95 (brs, 1H, H-4-DHP), 8.34 (brs, 1H, NH-DHP), 9.26 ppm (s, 2H, NH-amide). Molecular formula: C_25_H_36_ClN_5_O_2_; Calculated = C (63.34%) H (7.65%) N (14.77%); Found = C (63.40%) H (7.66%) N (14.81%).

*Computational studies*

The chemical structures of desired DHPs 3a-g were built and optimized using HyperChem software (version 7, Hypercube Inc.). Optimization of the compounds was performed through MM+ and PM3 methods and total energy gradient was calculated as a root mean square (RMS) value, until the RMS gradient was 0.01 kcal mol^-1^. The optimized conformer was transferred to Gaussian software to calculate HOMO, LUMO and partial atomic charge (Muliken) using RHF method and 3-21G basis set.

*In-vitro evaluation of anti-mycobacterial activity*

The test compounds 3a-g, were initially dissolved in DMSO to give a concentration of 1 or 2 mg/L. Except first column, all wells of micro plates were received 100 µL of freshly prepared Middle broke 7H9 medium (Himedia, India). 200 µL of distilled water was added to the first column of 96 well plates to minimize evaporation of the medium in the test wells during incubation. Then 100 µL of test compounds with desired concentrations (1000 or 2000 µL) were added to the wells of the first row (each concentration was assayed in duplicate) and serial dilution was made from the first row to the last. Microbial suspension of BCG (1173P2) (100 µL), which had been prepared with standard concentration of 0.5 McFarland and diluted with 1:10 proportion by the distilled water, was added to all test wells. Plates were then sealed and incubated for 4 days at 37°C. After that, 12µl Tween 80 10% and 20µl Alamar blue 0.01% (Himedia, India) were added to each test well. The results were assessed after 24 and 48 h. A blue color was interpreted as no bacterial growth, and color change to pink was scored as bacterial growth. Wells with a well-defined pink color were scored as positive for growth. The MIC (minimum inhibitory concentration) was defined as the lowest drug concentration, which prevented a color change from blue to pink. Ethambutol (Irandaru, Tehran) and DMSO were used as positive control and negative controls respectively (31).

## Results and Discussion

*Chemistry*

Seven new derivatives of dihydropyridine, compounds 3a-g (Figure 1), were synthesized using Hantzsch condensation in methanol at reflux condition and were purified by recrystallization in good yield (30% -85%). Structure of compounds was characterized by TLC followed by IR, elemental analysis and proton NMR.

*Computational studies*

Based on the subjects that mentioned in the introduction section, Partial atomic charge (PAC) of carbon atom of carbonyl moiety at the C-3 and C-5 position of dihydropyridine ring and the lipophilicity (log p) of DHPs 3a-3g was calculated using Gaussian and HyperChem software. To calculation the PAC, at first all of the compounds were optimized using HyperChem with molecular mechanics (MM+) and semi-empirical (PM3) methods. To finding the global minima, the best conformer from the previous stage was transferred to Gaussian and more optimization was performed using RHF method and 3-21G basis set.

 Confirmer with the global minima was used to calculation of partial atomic charges. Results of calculated PAC are presented in Table 2. Lipophilicity (log P) of desired compounds was calculated using HyeprChem (Table 2). Based on the results of the log p calculation (3d > 3e = 3f > 3c > 3b > 3a > 3g), the compounds 3d and 3g are the more and less lipophilic ligands respectively. So it might be concluded that the penetration of these ligands into the cell wall of mycobacterium is in order of 3d > 3e = 3f > 3c > 3b > 3a > 3g. Results of PAC calculation revealed in the compounds with aromatic substitution in the C-3 and C-5 (3a-3f), the carbon atom of carbonyl group at the C-3 position is more slightly positive than C-5 and result in to more susceptibility to bio hydrolytic activation. In the compound 3g which contains cycloalkyl group at the C-3 and C-5 position of DHP, the carbon atom of carbonyl group at the C-5 is more positive than C-3. Based on the results of mean of PAC at the C-3 and C-5, (3a > 3b > 3d > 3c > 3e > 3f > 3g), the carbonyl group of the compounds 3a and 3g is the more and less positive respectively. According to the PAC results that compound 3g has high value of PAC, it is expected that compound 3a is more susceptible to enzymatic hydrolysis to produce the active compound and so it should be more potent than compound 3g. 

**Table 2:** Calculated partial atomic charge (Muliken) of carbonyl groups at the C-3 and C-5 position of DHPs 3a-g using Gaussian software.

**compound**	**C-3 carbonyl partial charge**	**C-5 carbonyl partial charge**	**Meanof charge of C-3 & C-5**	**Logp** a
3a	0.955	0.903	0.929	0.43
3b	0.786	0.869	0.8275	0.80
3c	0.828	0.798	0.813	1.56
3d	0.822	0.823	0.8225	2.60
3e	0.843	0.798	0.8205	2.11
3f	0.824	0.771	0.7975	2.11
3g	0.748	0.806	0.777	0.33

a Calculated using HyperChem software.

*Anti-tubercular activity*

The ability of DHPs 3a-g to inhibition of mycobacterium tuberculosis growth was determined using in vitro assay. The results are summarized in the table 1. Each compound was dissolved in DMSO. Ethambutol and DMSO were used as positive and negative controls respectively. The in vitro screening data (Table 1) indicated that all analogs show a significant anti-tubercular activity in comparison to the reference drug ethambutol. Comparison of the MIC of compounds 3a, 3b, 3c and 3e ( 3a ≥ 3c > 3e > 3b) which contained the electron withdrawing groups (NO_2_, F, Cl and Br respectively) at the para position of phenyl ring, reveals that the compound 3a which contains more electronegative group is the more active than compounds 3c, 3e and 3b. Comparison of the MIC of compound 3e (4-Br) with 3f (3Br) and compound 3c (4-Cl) with 3d (3, 4-Cl), indicate that existence of the substitution at the 3 position of phenyl ring results in to reduce the activity, probably in order to their hindrances effect. 

 Comparison of compounds 3a-f with compound 3g reveals existence of the electron donating group (cyclohexyl) at C-3 and C-5 of DHP ring result in to reduce the activity probably in order to low values of partial atomic charge of carbonyl moieties. Based on the PAC, it was expected that compound 3b be more active than 3c, but experimental data was not confirmed that, may be because of their low partition coefficient (log P) and consequently low penetration into the mycobacterium cells.

## Conclusion

Seven DHPs analogs were synthesized and characterized by TLC, FT-IR and ^1^HNMR**. **The elemental analysis has confirmed the purity of products. The *in vitro* activities of all compounds against mycobacterium were investigated. Based on the *in vitro* screening data, all the designed and synthesized compounds (3a-3g) had good ability to inhibit mycobacterium tuberculosis growth in terms of MIC. The most potent compound was 3a, 4-nitrophenyl carboxamide derivative of DHPs, which was predicted in our computational studies based on PAC.

The results so far indicated that the activity of these ligands against the mycobacterium can significantly be influenced by log p of molecules and partial atomic charge of carbon atom of carbonyl moiety at C-3 and C-5 position of DHPs ring. Currently, our research group is exploring this idea to design newer ligands with better anti-tubercular activity.
